# Complicaciones de la otitis media con parálisis del sexto par craneal contralateral en pediatría

**DOI:** 10.7705/biomedica.5763

**Published:** 2021-06-15

**Authors:** Luz Miriam Leiva, Hamilton Delgado, Leidy Viviana Holguín, Christian Rojas

**Affiliations:** 1 Departamento de Medicina Física y Rehabilitación, Hospital Universitario del Valle, Universidad del Valle, Cali, Colombia Universidad del Valle Departamento de Medicina Física y Rehabilitación Hospital Universitario del Valle Universidad del Valle Cali Colombia; 2 Facultad de Ciencias de la Salud, Universidad Icesi, Cali, Colombia Universidad Icesi Facultad de Ciencias de la Salud Universidad Icesi Cali Colombia; 3 Facultad de Ciencias de la Salud, Pontificia Universidad Javeriana, Cali, Colombia Pontificia Universidad Javeriana Facultad de Ciencias de la Salud Pontificia Universidad Javeriana Cali Colombia; 4 Neurología Infantil, Departamento de Pediatría, Hospital Universitario del Valle, Universidad del Valle, Cali, Colombia Universidad del Valle Neurología Infantil, Departamento de Pediatría, Hospital Universitario del Valle Universidad del Valle Cali Colombia

**Keywords:** otitis media, hipertensión intracraneal, mastoiditis, petrositis, trombosis de seno intracraneal, enfermedades del nervio motor ocular externo, Otitis media, intracranial hypertension, mastoiditis, petrositis, sinus thrombosis, intracranial, *abducens nervus* diseases

## Abstract

La otitis media es una infección frecuente en la infancia, la cual puede producir complicaciones, incluidas las neurológicas graves, en cuatro de cada 100 niños en países en desarrollo.

Se presenta el caso de una niña de nueve años sin antecedentes de enfermedad que consultó por otitis media derecha, otorrea, síndrome de hipertensión intracraneal y parálisis del VI nervio craneal contralateral a la lesión. La tomografía computarizada de cráneo y la resonancia magnética cerebral revelaron otomastoiditis crónica, apicitis petrosa, y trombosis de los senos transverso y sigmoide, el bulbo yugular y la vena yugular interna derecha. Recibió tratamiento antibiótico y quirúrgico.

Este caso refleja el espectro de complicaciones intracraneales y extracraneales asociadas con la otitis media aguda en la era antibiótica. El examen físico permite la detección precoz de la hipertensión intracraneal, con signos como el papiledema y la parálisis del VI par contralateral como hallazgo inusual.

## Abstract (en)

Otitis media is a frequent infection during childhood. Complications may be present in up to 4 of 100 children including serious neurological complications, particularly in developing countries.

We report the case of a 9-year-old girl with no disease history who presented with otitis media, otorrhea, intracranial hypertension syndrome, and paralysis of the VI cranial nerve contralateral to the lesion. A computed tomography scan of the skull and a brain magnetic resonance imaging revealed chronic otomastoiditis, petrous apicitis, and thrombosis of the transverse and sigmoid sinus, the jugular bulb, and the right internal jugular vein. She received antibiotics and surgical treatment.

This case shows the spectrum of intra and extracranial complications associated with acute otitis media in the antibiotic era. The physical examination allows early identification of intracranial hypertension with signs such as papilledema and sixth contralateral nerve palsy as an unusual finding.

La otitis media aguda es una infección frecuente en la infancia (1) y, aunque se estima que sus complicaciones son menos frecuentes en la era antibiótica, estas pueden ser fatales (2,3). Cuatro de cada 100 niños con otitis media en los países en desarrollo presentan complicaciones (3).

Se reporta el caso de una niña con otitis media derecha complicada con colesteatoma, mastoiditis, apicitis petrosa, trombosis ipsilateral de los senos transverso y sigmoide con extensión a la yugular interna, síndrome de hipertensión intracraneal y parálisis del VI nervio craneal contralateral a la otitis.

## Presentación del caso

Se trata de una niña de nueve años de edad, migrante venezolana, residente en Colombia y sin afiliación al sistema de salud, que fue llevada al servicio de urgencias con fiebre de 40 °C de ocho días de evolución, cefalea, vómito, otalgia y otorrea derecha, sin síntomas respiratorios. Había recibido ampicilina e ibuprofeno ambulatoriamente durante cuatro días, sin prescripción médica. A los seis años fue sometida a amigdalectomía, tenía el esquema de vacunación completo para su edad, y no registraba otros antecedentes médicos.

El examen físico reveló otorrea derecha y eritema peritimpánico, y en la otoscopia izquierda no se observaron alteraciones. Se palpaban adenomegalias submandibulares y cervicales bilaterales menores de 1 cm y no tenía signos meníngeos. Se inició el tratamiento antibiótico con ampicilina-sulbactam por vía intravenosa (100 mg/kg por día).

En los exámenes de laboratorio de ingreso, se reportó leucocitosis de 22.000 por mm^3^, elevación de la proteína C reactiva en 58 mg/L, y no se registró disfunción hepática o renal.

**Figura 1:**
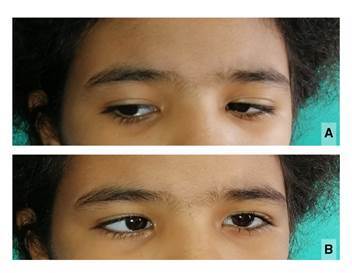
A) La mirada a la derecha es normal y, por lo tanto, el músculo recto lateral derecho se encuentra indemne. B) Se aprecia parálisis del músculo recto lateral izquierdo lo cual limita la mirada externa izquierda

Durante las primeras horas de observación en el servicio de urgencias, la paciente manifestó diplopía y, en el examen de oftalmología, se encontró restricción para la abducción del ojo izquierdo indicativa de parálisis del nervio motor ocular externo (*abducens nervus*) (figura 1). Además, el fondo de ojo evidenciaba papiledema bilateral, aunque no presentaba alteración sensitiva facial en la distribución del nervio trigémino, y la evaluación de los otros pares craneales fue normal.

Ante los signos de hipertensión intracraneal, se ordenó una tomografía computarizada de cráneo, simple y con contraste, la cual incluyó cortes axiales de 3 mm de espesor.

**Figura 2:**
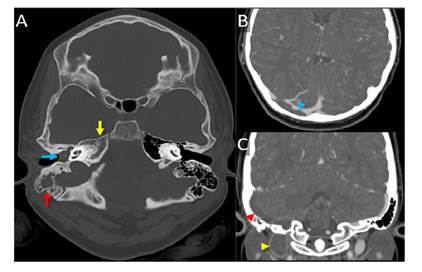
Tomografía computarizada de cráneo. **A.** Imágenes axiales en ventana ósea que muestran ocupación líquida de la cavidad timpánica (flecha azul) y de las mastoides (flecha roja) del lado derecho, así como alteración en la morfología y ocupación de las celdillas del ápex petroso derecho (flecha amarilla). **B.** y **C.** Imágenes axiales y coronales después del contraste que revelaron un defecto de opacificación central por trombosis aguda del seno transverso (cabeza de flecha azul), del sigmoide (cabeza de flecha roja) y del bulbo yugular (cabeza de flecha amarillo) del lado derecho.

En el lado derecho, se encontró trombosis aguda en el seno transverso que se extendía al agujero yugular y, hasta donde se veía, a la vena yugular interna. En los senos paranasales, se observó engrosamiento mucoso concéntrico de ambos senos maxilares y de las celdillas etmoidales, con nivel hidroaéreo en el seno maxilar del lado derecho y signos de sinusitis crónica agudizada. El engrosamiento del seno esfenoidal en el lado derecho era mínimo y se observaron secreciones que ocupaban todo el oído medio y las celdillas mastoideas del lado derecho, hallazgos que se relacionan con otomastoiditis aguda. La diferenciación de la sustancia gris y la sustancia blanca era adecuada y no se registraban lesiones intraaxiales ni extraaxiales, como tampoco realce anormal después de la administración del medio de contraste (figura 2).

Considerando que se presentaba una otitis media aguda complicada, se cambió el antibiótico a vancomicina (80 mg/kg por día) y ceftriaxona (100 mg/kg por día) y, dada la trombosis extensa de los senos durales, se inició anticoagulación con enoxaparina (1 mg/kg cada 12 horas).

**Figura 3:**
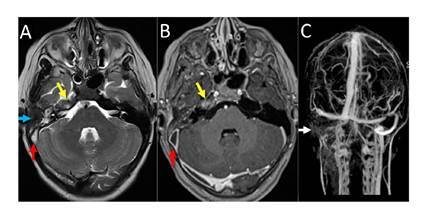
Resonancia magnética cerebral. **A.** y **B.** Cortes axiales ponderados en T2 y T1 después del contraste que revelaron cambios inflamatorios en el ápex petroso derecho (flecha amarilla) y en la cavidad timpánica (flecha azul) del lado derecho, indicativos de apicitis petrosa y otomastoiditis. Nótese la ocupación del seno sigmoide (flecha roja), por trombosis. **C.** Angiografía venosa en forma de “tiempo de vuelo” (time of flight, TOF) que evidenció ausencia de flujo en el seno transverso, el sigmoide y la vena yugular interna en el lado derecho, por trombosis venosa aguda (flechas blancas).

Los síntomas de hipertensión intracraneal persistían, por lo que en la Unidad de Neurocirugía Pediátrica se indicó administrarle acetazolamida (250 mg/día). Se practicó una resonancia magnética cerebral de 1,5 T en secuencias convencionales de forma simple y tras la administración de medio de contraste. Con este estudio se verificó una sinusitis crónica agudizada en ambos senos maxilares y etmoidales, y en el lado derecho, otomastoiditis aguda, apicitis petrosa, y trombosis de los senos sigmoides y transverso con extensión a la yugular interna; no había compromiso del seno cavernoso (figura 3).

Se hicieron estudios para descartar inmunodeficiencias primarias y secundarias, trombofilia o enfermedades autoinmunitarias. El ecocardiograma fue normal y la prueba de RT-PCR para el SARS-CoV2 fue negativa.

Los especialistas de Neurootología recomendaron la intervención quirúrgica; se optó por una resección amplia del temporal combinada con una técnica abierta, es decir, mastoidectomía radical y meatoconchoplastia.

Durante el procedimiento, se halló un colesteatoma en el oído medio con gran extensión al retrotímpano; el seno sigmoide se encontraba dehiscente en los tercios superior e inferior, con secreción purulenta. Ante estos hallazgos, los infectólogos decidieron continuar la administración de vancomicina, suspender la ceftriaxona e iniciar el cefepime (130 mg/kg/día). El tratamiento antibiótico y de anticoagulación se extendió hasta completar seis semanas.

**Figura 4:**
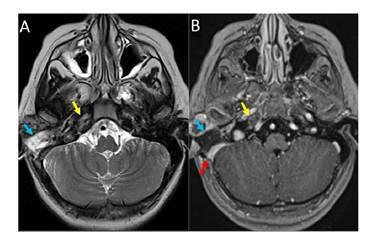
Resonancia magnética cerebral de control en el noveno día posquirúrgico. **A.** y **B.** Cortes axiales potenciados en T2 y T1 después del contraste, que evidenció cambios posquirúrgicos por mastoidectomía derecha (flecha azul) con disminución de los cambios inflamatorios en el ápex petroso (flecha amarilla). Nótese la recanalización parcial del seno sigmoideo derecho (flecha roja).

En la resonancia magnética cerebral con contraste realizada nueve días después del procedimiento quirúrgico, se evidenció regresión de los signos inflamatorios en las celdillas mastoideas y el ápex petroso, y recanalización de la trombosis en los senos venosos y la yugular interna (figura 4). Además, hubo resolución de los síntomas de hipertensión intracraneal. Sin embargo, a los dos meses del ingreso hospitalario la parálisis del VI nervio craneal persistía.

### Consideraciones éticas

Se obtuvo el consentimiento informado de los padres de la paciente y el aval del Comité Institucional de Revisión de Ética Humana de la Universidad del Valle, CIREH (código RC 005-020).

## Discusión

Se presenta el caso de una paciente de nueve años con un amplio espectro de complicaciones intracraneales por otitis media aguda, como hipertensión intracraneal y trombosis de los senos durales, y complicaciones extracraneales, como colesteatoma, mastoiditis crónica, apicitis petrosa y trombosis venosa yugular.

La alta prevalencia de otitis media aguda en la infancia y sus potenciales complicaciones (4) requieren el examen neurológico completo de rutina para detectar el compromiso de los pares craneanos y la presencia de meningitis e hipertensión intracraneal (3,5,6).

En los pacientes con signos clínicos de compromiso neurológico, se requieren estudios de neuroimágenes (5) para establecer tempranamente si hay complicaciones intracraneales y extracraneales (7). La tomografía computarizada es el estudio inicial de elección para evaluar la afectación ósea y extracraneal, así como la mayoría de las complicaciones intracraneales. La resonancia magnética cerebral se aconseja para una mejor localización y descripción de las complicaciones intracraneales (8), hallazgos que son relevantes para optar por el tratamiento conservador o el quirúrgico.

El síndrome de Gradenigo es una rara complicación de la otitis media en la era antibiótica (9), el cual se caracteriza por otorrea, parálisis del VI par craneal ipsilateral y dolor en la inervación facial del V par craneal debido a la otitis media que compromete el ápex petroso (5); también, se ha reportado trombosis de los senos venosos asociada con este síndrome (5,10) y papiledema bilateral (11).

En este caso, la paciente presentaba otitis media con otorrea, parálisis del VI par craneal (contralateral), aunque sin alteración sensitiva en el territorio del nervio trigémino. En la resonancia magnética cerebral se confirmó la presencia de otomastoiditis, apicitis petrosa y trombosis ipsilateral de los senos venosos cerebrales con extensión a la vena yugular interna.

Este caso puede confundirse fácilmente con el síndrome de Gradenigo, sin embargo, no toda apicitis petrosa es indicativa del síndrome. Recientemente, se definieron los criterios clasificatorios de este síndrome en tres categorías: clásica, incompleta y mimética (5). Con base en estas, se pudo aclarar que este caso no cumplía con los criterios diagnósticos del síndrome de Gradenigo, pues no basta con la presencia de apicitis petrosa, sino que debe acompañarse de parálisis del VI par craneal ipsilateral o dolor facial en la zona inervada por el nervio trigémino, lo que estaba ausente en esta paciente.

En el síndrome de Gradenigo, la parálisis del VI par es ipsilateral a la apicitis petrosa; también, se han descrito casos de compromiso bilateral del VI par (5,12), pero no así de compromiso contralateral del nervio motor ocular externo. En un reporte de caso reciente que describe las manifestaciones oculares del síndrome, si bien se informa de la presencia de papiledema bilateral, la parálisis del VI par es constantemente ipsilateral con respecto a la otomastoiditis (11).

El compromiso de este nervio se explica por el contacto del ápex petroso con la porción del VI par craneal dentro del canal de Dorello en su trayecto por debajo del ligamento petroclival, que está expuesto a procesos inflamatorios (13). Además, es importante estudiar el mimetismo del síndrome de Gradenigo (5) y otras causas de parálisis del nervio motor ocular externo.

En este caso, se descartó trombofilia e inmunodeficiencias, y la parálisis del VI par se atribuyó a la hipertensión intracraneal, que puede desplazar el tronco encefálico hacia abajo, provocando el estiramiento excesivo del nervio *abducens* desde su emergencia, cuando atraviesa la cisterna prepontina (13).

En este caso, se presentó trombosis de la vena yugular interna, sin embolia séptica y sin que se hubiera documentado la presencia de algún microorganismo causal, razón por la cual no calificaba como síndrome de Lemierre de origen otogénico (14,15).

Entre las limitaciones del reporte, debe mencionarse el que no se tomaron hemocultivos, pues la paciente recibió antibiótico ambulatoriamente y desde el ingreso hospitalario, por lo tanto, no se aisló ningún microorganismo. Tampoco, se practicó una punción lumbar al considerar que existía riesgo por la extensa trombosis y por la anticoagulación. Por otra parte, no se pudo hacer el seguimiento ambulatorio de la paciente por su condición de migrante y el hecho de no estar afiliada al sistema de salud.

## Conclusión

Este caso ilustra un espectro de complicaciones intracraneales y extracraneales de la otitis media aguda en niños en la era antibiótica. Se recomienda hacer un interrogatorio y un examen físico completo que permitan la detección precoz de la hipertensión intracraneal, con signos como el papiledema y la parálisis del VI par contralateral como hallazgo inusual. Además, deben practicarse estudios de neuroimágenes con contraste en aquellos pacientes con manifestaciones neurológicas, para detectar las complicaciones potencialmente fatales de la otitis media aguda.
